# Is a ‘guideline-compliant’ primary cesarean delivery associated with a modified risk for maternal and neonatal morbidity?: a clinical evaluation of the 2014 ACOG/SMFM obstetric care consensus statement

**DOI:** 10.1186/s12884-021-04048-1

**Published:** 2021-08-22

**Authors:** Andrew W. White, Charis N. Chambers, Michelle C. Ertel, Taylor R. Gennaro, Ling Chen, Alexander M. Friedman, Kacey Y. Eichelberger

**Affiliations:** 1 Department of Obstetrics and Gynecology, Prisma Health Upstate/University of South Carolina School of Medicine Greenville, West Faris Road, Suite 470, Greenville, SC 29605 USA; 2 Division of Pediatric and Adolescent Gynecology, Department of Obstetrics and Gynecology, Baylor University, 1 Baylor Plaza, Houston, TX 77030 USA; 3 Department of Obstetrics and Gynecology, University of North Carolina Chapel Hill, 3009 Old Clinic Building CB 7570, Chapel Hill, NC 27599 USA; 4 University of South Carolina School of Medicine, 6311 Garners Ferry Road, Columbia, SC 29209 USA; 5 Department of Obstetrics and Gynecology, Columbia University, 622 W 168th St, New York, NY 10032 USA; 6 Division of Maternal-Fetal Medicine, Department of Obstetrics and Gynecology, Columbia University, 622 W 168th St, New York, NY 10032 USA; 7 Division of Mater nal-Fetal Medicine, Department of Obstetrics and Gynecology, Prisma Health Upstate/University of South Carolina School of Medicine Greenville, 890 West Faris Road, Suite 470, Greenville, SC 29605 USA

**Keywords:** Cesarean delivery, Maternal morbidity, Neonatal morbidity, Guidelines

## Abstract

**Background:**

It is currently unknown whether primary CDs performed in compliance with the 2014 ACOG/SMFM Obstetric Care Consensus Statement guidelines (“guideline-compliant”) are associated with a modified risk of maternal and neonatal morbidity, when compared to primary CDs performed outside the guidelines (“guideline-noncompliant”). Our primary objective was to determine if a guideline-compliant primary CD is associated with a modified risk for maternal or neonatal morbidity, when compared to guideline-noncompliant primary CD.

**Methods:**

A retrospective cohort study of all primary CDs at one tertiary referral center in the calendar year following publication of the Consensus Statement. Logistic regression was performed to calculate the risk of adverse maternal and neonatal outcomes for guideline-compliant primary CDs, when compared to guideline-noncompliant and guideline-not addressed, and when adjusted for maternal age, BMI, hypertension, gestational age at delivery, insurance carrier, and provider practice.

**Results:**

Eight hundred twenty-seven primary CDs were included during the study period, of which 34.8, 26.0, and 39.2% were guideline compliant, guideline-noncompliant, and guideline-not addressed. No statistically significant differences in the frequency of adverse maternal outcomes across these three groups were observed with the exception of maternal ICU admission, which was significantly associated with a guideline-not addressed primary CD (*p* = 0.0002). No statistical difference in rates of NICU admissions, 5 min APGAR < 5, or umbilical artery cord pH < 7 were observed between guideline-compliant and guideline-noncompliant primary CDs.

**Conclusion:**

Women undergoing guideline-compliant primary CDs were not significantly more likely to experience a maternal or neonatal morbidity when compared to guideline-noncompliant primary CDs.

## Background

From 1996 to 2009 the rate of cesarean delivery (CD) in the United States increased from 20.7 to 32.9%, and has since plateaued at 31.9% in 2018 [1]. While life-saving in many cases, CD is associated with an increase in maternal morbidity, maternal mortality, and neonatal morbidity, when compared to vaginal delivery (VD) [2]. A history of CD is associated with increased risk for future CD, and increased morbidity in subsequent pregnancies [3].

The ideal CD rate to minimize maternal and neonatal morbidity and mortality is not well established. In 2014, both the American College of Obstetricians and Gynecologists (ACOG) and the Society for Maternal Fetal Medicine (SMFM) issued a national call to reduce the primary CD rate, codified in the Obstetric Care Consensus Statement, “Safe Prevention of the Primary Cesarean Delivery.” [3] In this joint document, these national organizations outlined clear and specific recommendations for the management of the following, each of which are linked to a common indication for primary CD: first and second stages of labor, fetal heart rate monitoring, induction of labor, fetal malpresentation, suspected fetal macrosomia, excessive maternal weight gain, and twin gestations. Evidence suggests compliance with these recommendations in clinical practice is associated with a reduction in the primary CD rate [4].

With any introduction of clinical care guidelines, ongoing evaluation of actual clinical outcomes is critical to determine (a) if implementation of the guidelines in ‘real life’ settings results in the outcome intended, and (b) to determine if compliance with the guidelines is associated with any unanticipated adverse outcomes. For example, ACOG had advocated for a reduction in non-medically indicated early-term deliveries for more than 10 years, a policy recommendation frequently referred to as “the 39 week rule.” [5, 6] While implementation of this recommendation has been positively associated with a reduction in both total number of early term deliveries and neonatal morbidity [7], some data support an unanticipated increased risk of stillbirth at term [8, 9].

It is currently unknown whether primary CDs performed in compliance with the 2014 Obstetric Care Consensus Statement guidelines (“guideline-compliant”) are associated with a modified risk of maternal and neonatal morbidity, when compared to primary CDs performed outside the new guidelines (“guideline-noncompliant”). Our primary objective was to determine if a guideline-compliant primary CD is associated with a modified risk for maternal or neonatal morbidity, when compared to guideline-noncompliant primary CD. We hypothesized that guideline-compliant CDs are associated with increased maternal morbidity, when compared to guideline-noncompliant primary CDs, as guideline-compliant CDs for failed induction of labor or arrest of the first or second stages are associated with a longer window from admission-to-delivery.

## Methods

This retrospective cohort study included all women undergoing primary CD at a single tertiary referral center between January 1 and December 31, 2015, the first full calendar year following publication of the Obstetric Care Consensus Statement guidelines. During the previous year, our department embarked on substantial quality improvement (QI) work around the Consensus Statement, which included educational sessions for all delivery providers and labor unit nurses, simulation work, and the public posting of the guidelines on all Labor and Delivery units. While compliance with the guidelines was not formally enforced, it was strongly encouraged and modeled by physician and nursing leadership in a continuous QI process.

Eligible subjects were identified by a query of SoftMed® for the ICD-9 code 654.21 and the ICD-10 code 03421 (primary CD). The total number of women delivering in the same time period without a history of prior CD was also identified by a SoftMed query; this number was used to calculate primary CD rates (defined as the number of women delivering via primary CD in a given time frame divided by the number of women delivering during the same time frame without a prior CD).

Basic demographic data was collected including maternal age, race/ethnicity, insurance payer and marital status. Clinical covariates of interest included BMI (kg/m2 at initiation of prenatal care), parity, gestational age at delivery, single versus multiple gestations, and labor status on admission (spontaneous, induced, augmented, or none). Presence of the three most prevalent medical co-morbidities in our patient population was evaluated: smoking status, hypertension and diabetes mellitus. Variables were collected from manual chart review from the electronic medical records systems used at our institution during the study period, which include Soarian, Centricity Perinatal, Sovera, and Epic.

The indication for a primary CD was identified from reviewing the operative report, the archived labor notes, and any additional clinical documentation. This detailed chart review was performed by one of two physician investigators (AW, CC). Each primary CD was then classified into one of three study groups (“guideline-compliant,” “guideline-noncompliant,” or “guideline-not addressed”) by comparing provider and/or labor nurse documentation to a standard rubric crafted from the 2014 Obstetric Care Consensus Statement, “Safe Prevention of the Primary Cesarean Delivery” (Table 1) [3]. Complex cases were adjudicated by a single senior investigator (KE) at our institution.

**Table 1:** Guidelines for “compliant” cesarean deliveries

**Failed induction of labor**	Latent phase persists despite: • Cervical ripening for Bishops score ≤ 6 cm • At least 24 h in the latent phase (defined as from initiation of cervical ripening, pitocin started, or AROM [whichever came first] to time of delivery) • At least 12 h of pitocin after rupture of membranes
**Arrest of the first stage of labor**	Cervix ≥6 cm and ruptured membranes with: • No cervical change despite 4 h of adequate uterine contractions with IUPC and MVU > 200 Or • No cervical change despite 6 h of inadequate uterine contractions with or without IUPC
**Arrest of the second stage of labor**	• Operative VD attempted for arrest and unsuccessful Or • At least 2 h of pushing in multiparous women Or • At least 3 h of pushing in nulliparous women And • If vertex is documented as malpositioned, manual rotation of fetal occiput must be attempted (only compliant for c/s if also pushed for the above durations)
**Macrosomia**	• Ultrasound estimated fetal weight ≥ 4500 g in women with diabetes Or • Ultrasound estimated fetal weight ≥ 5000 g in women without diabetes
**Malpresentation**	• External cephalic version attempted and failed Or • External cephalic version counseling documented and declined
**Twin gestations**	• Presenting twin is cephalic and patient counseled toward VD but opted for CD Or • Presenting twin is non-cephalic
**Non-reassuring fetal heart tones**	• Amnioinfusion prior to CD in the setting of variable decelerations And/Or • Scalp stimulation documented prior to CD in the setting of minimal or absent variability

Based on the ACOG/SMFM 2014 Obstetric Care Consensus Statement, “Safe Prevention of the Primary Cesarean Delivery” [3]

Adverse maternal outcomes included: chorioamnionitis; postpartum hemorrhage, defined as an estimated blood loss > 1000 mL [10]; maternal infectious complications (superficial surgical site infection [involvement of skin or subcutaneous tissue] [11], deep incisional surgical site infection [involvement of fascia and muscle] [11], organ/space surgical site infection [11], endometritis, urinary tract infection, or pneumonia); surgical complications (hysterectomy, broad ligament hematoma, cystotomy [requiring repair], ureteral injury, bowel injury [requiring repair]); reoperation; and transfusion of packed red blood cells. Adverse neonatal outcomes included NICU admission, 5 min Apgar ≤5, umbilical artery cord gas pH ≤ 7, and neonatal death.

Statistical analysis was performed with SAS Statistical Software (Cary, North Carolina). Chi squared and Fisher’s exact test were used for categorical data, where appropriate, and t-tests were used for continuous variables. Logistic regression was performed to calculate the risk of adverse maternal and neonatal outcomes for guideline-compliant primary CDs, when compared to guideline-noncompliant and guideline-not addressed, and when adjusted for maternal age, BMI, hypertension, gestational age at delivery, insurance carrier, and provider practice. A two-sided *p* value < 0.05 was considered statistically significant. This study conforms to STROBE guidelines for cohort studies.

## Results

Our final cohort included 827 women who delivered via primary CD during the study period. During the same period, 4938 women delivered without a history of prior CD, for a total institutional primary CD rate of 16.7%. Of our total primary CD cohort, 288 (34.8%) were determined to be guideline-compliant, 215 (26.0%) were guideline-noncompliant, and 324 (39.2%) were guideline-not addressed.

Baseline demographics of these three study groups are listed in Table 2. Statistically significant differences identified between the study groups include maternal parity, gestational age at delivery, labor status, and number of fetuses.

**Table 2:** Baseline demographics

	Compliant		Non-compliant		Not addressed		
	N	(%)	N	(%)	N	(%)	*p*-value
**All**	288	(34.8)	215	(26.0)	324	(39.2)	
**Maternal age**							0.09
14–17	3	(1.0)	4	(1.9)	7	(2.2)	
18–24	84	(29.2)	49	(22.8)	82	(25.3)	
25–29	85	(29.5)	63	(29.3)	78	(24.1)	
30–34	75	(26.0)	70	(32.6)	92	(28.4)	
35–39	30	(10.4)	25	(11.6)	42	(13.0)	
> 39	11	(3.8)	4	(1.9)	23	(7.1)	
**Race/Ethnicity**							0.27
Asian	5	(1.7)	6	(2.8)	9	(2.8)	
Black	61	(21.2)	31	(14.4)	74	(22.8)	
White	193	(67.0)	151	(70.2)	203	(62.7)	
Hispanic	24	(8.3)	24	(11.2)	36	(11.1)	
Other/unknown	5	(1.7)	3	(1.4)	2	(0.6)	
**Payer**							< 0.001
Commercial	156	(54.2)	151	(70.2)	160	(49.4)	
Medicaid	129	(44.8)	57	(26.5)	152	(46.9)	
Medicare	0	(0.0)	1	(0.5)	4	(1.2)	
Self pay	0	(0.0)	2	(0.9)	5	(1.5)	
Other	3	(1.0)	4	(1.9)	3	(0.9)	
**Marital status**							0.05
Married	150	(52.1)	137	(63.7)	174	(53.7)	
Not married	137	(47.6)	76	(35.3)	149	(46.0)	
Unknown	1	(0.3)	2	(0.9)	1	(0.3)	
**BMI**							0.29
< 20	14	(4.9)	9	(4.2)	23	(7.1)	
20- < 25	70	(24.3)	52	(24.2)	91	(28.1)	
25- < 30	83	(28.8)	65	(30.2)	73	(22.5)	
30- < 35	61	(21.2)	35	(16.3)	63	(19.4)	
>/=35	60	(20.8)	54	(25.1)	74	(22.8)	
**Para**							< 0.001
0	227	(78.8)	190	(88.4)	219	(67.6)	
1	35	(12.2)	12	(5.6)	59	(18.2)	
> 1	26	(9.0)	13	(6.0)	46	(14.2)	
**Gestational age**							< 0.001
< 32	8	(2.8)	3	(1.4)	55	(17.0)	
32 to < 37	27	(9.4)	21	(9.8)	79	(24.4)	
37 to < 39	73	(25.3)	44	(20.5)	70	(21.6)	
39 to < 41	133	(46.2)	122	(56.7)	108	(33.3)	
>/=41	47	(16.3)	25	(11.6)	12	(3.7)	
**Labor**							< 0.001
Augmented	23	(8.0)	24	(11.2)	18	(5.6)	
Induced	109	(37.8)	107	(49.8)	73	(22.5)	
Spontaneous	69	(24.0)	40	(18.6)	77	(23.8)	
None	87	(30.2)	44	(20.5)	156	(48.1)	

Based on the 2014 Guidelines, indications for primary CD were divided into six categories (Table 1). During the study period, labor arrest (including failed induction and arrests of the first and second stages of labor) was the most common indication for primary CD, followed by (in order): non-reassuring fetal tracing; malpresentation; “other” (which included: prior myomectomy, placenta/vasa previa, cord prolapse, maternal infections [HIV with viral load > 1000 or active HSV], presumed fetal intolerance of labor, prior 3rd or 4th degree perineal laceration, prior shoulder dystocia, fetal anomaly, maternal request, maternal condition, or fetal condition); multiple gestations; and macrosomia.

The frequency of adverse maternal outcomes associated with guideline-compliant versus guideline-noncompliant and guideline-not addressed primary CDs are shown in Table 3. There were no statistically significant differences in the frequency of adverse maternal outcomes across these three groups with the exception of maternal ICU admission, which was significantly associated with a guideline-not addressed primary CD (*p* = 0.0002).

**Table 3:** Maternal outcomes

	Compliant		Noncompliant		Not addressed		
	N	(%)	N	(%)	N	(%)	*p*-value
**All patients**	288	(34.8)	215	(26.0)	324	(39.2)	
**Maternal outcomes**							
Maternal death	0	0.00%	0	0.00%	1	0.31%	0.46
VTE	1	0.35%	0	0.00%	2	0.62%	0.51
ICU admission	0	0.00%	0	0.00%	11	3.40%	0.0002
Transfusion	11	3.82%	5	2.33%	21	6.48%	0.06
PPH	7	2.43%	10	4.65%	13	4.01%	0.38
**Surgical complication**							
Reoperation	1	0.35%	1	0.47%	2	0.62%	0.89
Bowel injury	0	0.00%	0	0.00%	1	0.31%	0.46
Ureteral injury	0	0.00%	0	0.00%	0	0.00%	N/A
Cystotomy	0	0.00%	1	0.47%	0	0.00%	0.24
Broad ligament hematoma	0	0.00%	1	0.47%	1	0.31%	0.55
Hysterectomy	0	0.00%	0	0.00%	3	0.93%	0.10
**Maternal infections**							
Pneumonia	0	0.00%	0	0.00%	1	0.31%	0.46
Urinary tract infection	2	0.69%	0	0.00%	2	0.62%	0.49
Endometritis	8	2.78%	4	1.86%	4	1.23%	0.38
Superficial SSI	3	1.04%	2	0.93%	3	0.93%	0.99
Deep incisional SSI	0	0.00%	1	0.47%	0	0.00%	0.24
Organ/space SSI	0	0.00%	0	0.00%	0	0.00%	N/A

Adverse neonatal outcomes associated with primary CD during the study period are presented in Table 4. The risk of neonatal death, low pH < 7, and NICU admission was significantly higher with guideline-not addressed primary CD, when compared to guideline-compliant and guideline-noncompliant primary CDs (*p* = 0.009, 0.021, and < 0.001, respectively). When guideline-not addressed primary CDs were excluded from the analysis, there was no statistical difference in rates of NICU admissions, 5 min APGAR < 5, or umbilical artery cord pH < 7 between guideline-compliant and guideline-noncompliant primary CDs.

**Table 4:** Neonatal outcomes

	Compliant		Noncompliant		Not addressed		
	N	(%)	N	(%)	N	(%)	*p*-value
**All patients**	288	(34.8)	215	(26.0)	324	(39.2)	
**Neonatal outcomes**							
NICU admission	51	17.71%	27	12.56%	148	45.68%	< 0.0001
Low Apgar (≤5)	11	3.82%	5	2.33%	21	6.48%	0.06
Low pH (≤7)	3	1.04%	1	0.47%	11	3.40%	0.02
Neonatal death	0	0.00%	0	0.00%	9	2.78%	0.0009

In logistic regression analyses, women undergoing guideline-compliant primary CD were no more likely to experience an adverse maternal outcome when compared to guideline-noncompliant and guideline-not addressed primary CDs. This remained true when adjusted for maternal parity, gestational age at delivery, labor status, and number of fetuses (AOR 1.10, 95% CI 0.58–2.07; AOR 1.20, 95%CI 0.67–2.14, respectively). However, guideline not-addressed primary CDs were associated with a significantly increased risk of any adverse maternal or neonatal outcomes, when compared to guideline-compliant primary CDs and when adjusted for maternal parity, gestational age at delivery, labor status, and number of fetuses (AOR 4.16, 95% CI 2.78–6.23).

Finally, a sensitivity analysis was performed for the logistic regression model including any maternal or neonatal morbidity but restricted to births ≥37 weeks. Guideline-not addressed primary CDs remained significantly associated with an increased but attenuated risk for either maternal or neonatal morbidity when compared to guideline-compliant primary CDs (AOR1.95, 95% CI 1.18–3.22).

## Discussion

This quality improvement work suggests that over the course of 1 year at a tertiary referral center, women undergoing guideline-compliant primary CDs were not significantly more likely to experience a maternal or neonatal morbidity when compared to guideline-noncompliant primary CDs. Therefore, it is plausible that compliance with the guidelines recommended in the ACOG/SMFM 2014 Obstetric Care Consensus Statement, “Safe Prevention of the Primary Cesarean Delivery” [3] may be associated with a decreased primary CD rate without an increased risk of adverse maternal or neonatal outcomes. This hypothesis should be tested in larger cohorts, as we were underpowered to detect small differences in more rare outcomes given our fixed sample size. In post hoc analysis, we estimate that a future cohort would need a sample size of 14,160 primary CDs to detect a significant difference in frequency of transfusion (the most prevalent adverse maternal outcome in our study) between guideline-compliant and guideline-noncompliant primary CDs, if one exists.

This work represents the sum of a year-long Quality Improvement (QI) initiative in our large academic medical center. After the publication of the 2014 Consensus Statement, we conducted multiple educational sessions for all delivery providers and nurses on the topic and posted the new rubric in highly visible locations on our Labor and Delivery units. While individual obstetric providers were not formally required to follow the guidelines (as evidenced by a 26% rate of guideline noncompliance), there was strong cultural and institutional influence to follow the new guidelines. After so much intentional focus on guideline compliance versus noncompliance, a very unexpected finding was that the largest percentage of our cohort (39%) underwent guideline-not addressed primary CD. Therefore, the Obstetric Care Consensus Statement recommendations, at most, only apply to 61% of our study population. We hypothesize that the higher rates of any maternal or neonatal adverse outcome associated with guideline-not addressed primary CDs when compared to guideline-compliant or guideline-noncompliant may be attributable to confounding by indication, as many of these women had indications for due to confounding by indication; indeed for many of those cases, it was the indication for CD that predisposed them to maternal ICU admission, NICU admission, or low 5 min APGAR, not the CD itself.

Strengths of our study include that this was the first clinical evaluation of primary CD morbidity following publication of the ACOG/SMFM 2014 Obstetric Care Consensus Statement, “Safe Prevention of the Primary Cesarean Delivery,“ [3] a critical step after implementation of any new clinical care guidelines in real practice settings. Furthermore, data on clinical practice and decision-making (eg, number of hours spent in each labor phase and stage, number of hours exposed to oxytocin, whether or not external cephalic version was offered) were collected by one of two physicians in granular detail after thorough chart review. This quality of data cannot be easily extracted from databases alone and represents a unique strength of our approach. This hypothesis generating work should be replicated in larger longitudinal cohorts to better define excess risk associated with guideline compliance, if any exists.

A weakness of this study is that it was performed at a single tertiary referral institution as part of internal QI work, and the sample size was inherently limited by delivery volume over that 12 month study window. As this was a pragmatic QI design and formal sample size analysis was not done a priori, we were underpowered to detect small differences in rare outcomes. That said, our data are hypothesis generating and can inform power calculations for future investigations on this topic. Furthermore, while we controlled for the three most common comorbidities in our population (hypertension, diabetes, and smoking), it is possible other maternal medical comorbidities impact the association between guideline compliance and either maternal or neonatal outcomes. We were also unable to quantify the impact of delivery provider type (physician versus midwife) on compliance with ACOG guidelines given the small number of midwifery providers in our sample.

## Conclusion

Providers and institutions have done meaningful work to safely reduce the rate of CD in the US, including systematic approaches to scaling the ACOG/SMFM 2014 Obstetric Care Consensus Statement, “Safe Prevention of the Primary Cesarean Delivery, and the Council on Patient Safety in Women’s Health Care’s “Patient Safety Bundle on the Safe Reduction of Primary Cesarean Births” across multiple sites [12–14]. That said, the primary outcome for this sort of work is usually, and predictably, the rate of CD. While reducing that absolute rate is important, we must remain mindful of the larger goal: the lowest possible rate of aggregate maternal and neonatal morbidity. For our patients’ safety, critically analyzing the downstream consequences of nationally recommended safety bundles and obstetric care consensus statements will be as important as our analysis of their primary efficacy.

## Data Availability

The datasets used and/or analysed during the current study are available from the corresponding author on reasonable request.

## Abbreviations

ACOG: American College of Obstetricians and Gynecologists
SMFM: Society for Maternal Fetal Medicine
CD: Cesarean delivery
ICU: Intensive care unit
NICU: Neonatal intensive care unit
